# Incomplete Multiview Clustering via Late Fusion

**DOI:** 10.1155/2018/6148456

**Published:** 2018-10-01

**Authors:** Yongkai Ye, Xinwang Liu, Qiang Liu, Xifeng Guo, Jianping Yin

**Affiliations:** ^1^College of Computer, National University of Defense Technology, Changsha, China; ^2^Dongguan University of Technology, Dongguan, China

## Abstract

In real-world applications of multiview clustering, some views may be incomplete due to noise, sensor failure, etc. Most existing studies in the field of incomplete multiview clustering have focused on early fusion strategies, for example, learning subspace from multiple views. However, these studies overlook the fact that clustering results with the visible instances in each view could be reliable under the random missing assumption; accordingly, it seems that learning a final clustering decision via late fusion of the clustering results from incomplete views would be more natural. To this end, we propose a late fusion method for incomplete multiview clustering. More specifically, the proposed method performs kernel *k*-means clustering on the visible instances in each view and then performs a late fusion of the clustering results from different views. In the late fusion step of the proposed method, we encode each view's clustering result as a zero-one matrix, of which each row serves as a compressed representation of the corresponding instance. We then design an alternate updating algorithm to learn a unified clustering decision that can best group the visible compressed representations in each view according to the *k*-means clustering objective. We compare the proposed method with several commonly used imputation methods and a representative early fusion method on six benchmark datasets. The superior clustering performance observed validates the effectiveness of the proposed method.

## 1. Introduction

The term “multiview data” refers to a collection of different data sources or modalities that describe the same samples. For example, clinical text and images serve as two views of a patient's diagnosis file, or an image on a webpage may be described by the pixel data and the surrounding text. Clustering is one of the unsupervised learning tasks that divides samples into disjointed sets, revealing the intrinsic structure of the samples [1–3]. Multiview clustering aims to utilize the information from various views for better clustering performance. A number of studies have been conducted to explore multiview clustering; these studies can be roughly divided into two categories. The methods in the first category create a fusion of the multiview information in the early stage and then perform clustering [4–6]. The methods in the second category group samples in each view and then create a late fusion of the clustering results from different views to obtain the final clustering decision [7, 8].

However, in real-world applications of multiview clustering, incomplete views often exist. For example, in patient grouping [9], patients often undergo various tests, but some patients may fail to undergo particular tests due to poor health or the high costs involved. Alternatively, in user grouping for a recommendation system [10], a user's multiview data consists of transaction histories, social network information, and credit records from different systems; however, it is not guaranteed that all users will have complete information from all systems.

A straightforward strategy for handling incomplete multiview clustering is to first fill the incomplete view information and then apply the common multiview clustering algorithm. Some widely used filling algorithms include zero filling, mean value filling, and *k*-nearest neighbor filling.

In addition to simple filling methods, a few early fusion methods have been proposed for incomplete multiview clustering. In [11], a method was proposed to deal with cases where one view is complete and the other is incomplete. The kernel matrix of the incomplete view is imputed following Laplacian regularization from the complete view. Kernel canonical correlation analysis is then performed to ascertain the projected space that maximizes the correlation between the corresponding projected instances across the two views. Based on this work, a method was proposed to solve the problem when the two views are incomplete [10]. This method iteratively updates the kernel matrix of one view using Laplacian regularization from the other view. Using this work as a foundation, Zhao et al. [12] added global graph regularization of the samples to guide the learning of the subspace. A similar work proposed to integrate the feature learning process without the nonnegative constraints on the data [13]. However, all of the above works are either limited to two views or hard to adapt to more than two views. Recently, Shao et al. [14] proposed a multiview clustering method not limited to two views. The proposed method learns the latent representations in subspace for all views, then produces a consensus representation that minimizes the difference between views, after which clustering is performed on the consensus representation.

What these studies overlook is that the clustering results from the incomplete views could be reliable under a random missing assumption. Most of the studies on incomplete multiview clustering are based on this assumption, which holds that whether an instance in a view is missing is not relevant to the corresponding sample's cluster label. Under this assumption, the missing ratios of each cluster should be almost the same; therefore, the overall cluster structure could be kept in an incomplete view.

**Figure 1:**
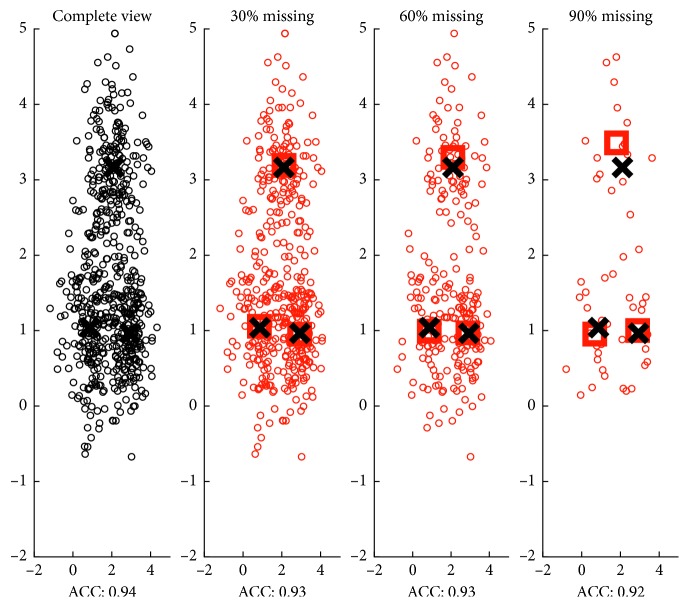
Cluster structure of the visible instances remains stable when this view suffers different ratios of random missing. The complete view consists of three Gaussian distributions. ACC is the kernel- (*k-*) means clustering accuracy of the visible instances. Black crosses are cluster centroids of the complete view. Red squares are cluster centroids of the visible instances under random missing.

**Figure 2:**
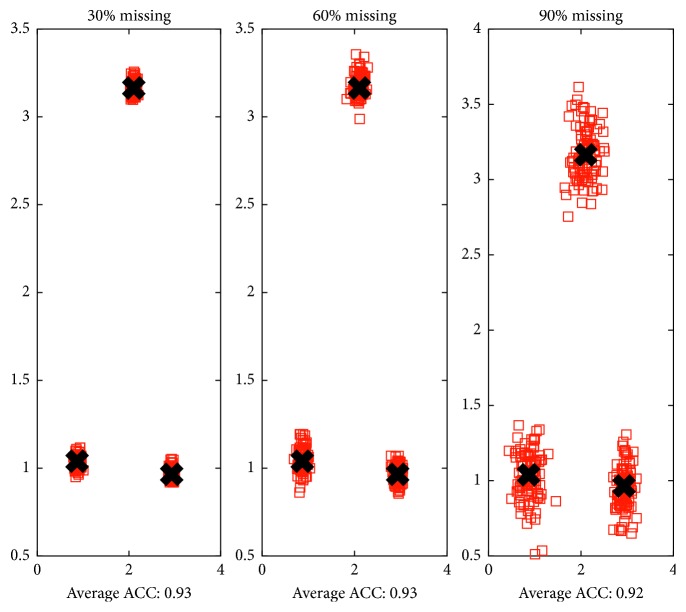
We repeat the random missing procedure for 100 times at different missing ratios. We calculate the average ACC and plot the cluster centroids of the visible instances under random missing. Black crosses are cluster centroids of the complete view. Red squares are cluster centroids of the visible instances under random missing.

Accordingly, we build a toy data consisting of three Gaussian distributions to illustrate how the cluster structure could be maintained under random missing conditions. We randomly delete the instances with different ratios and perform kernel *k*-means on the visible instances. From Figure 1, it can be observed that the clustering accuracy (ACC) of the visible instances is stable when the missing ratio increased; moreover, the cluster centroids of the visible instances under random missing stay near the cluster centroids of the complete view. Moreover, we repeat the random missing procedure for 100 times at different missing ratios. As shown in Figure 2, the average ACC of the visible instances also remain stable, and the cluster centroids of the visible instances stay around the cluster centroids of the complete view.

**Figure 3:**
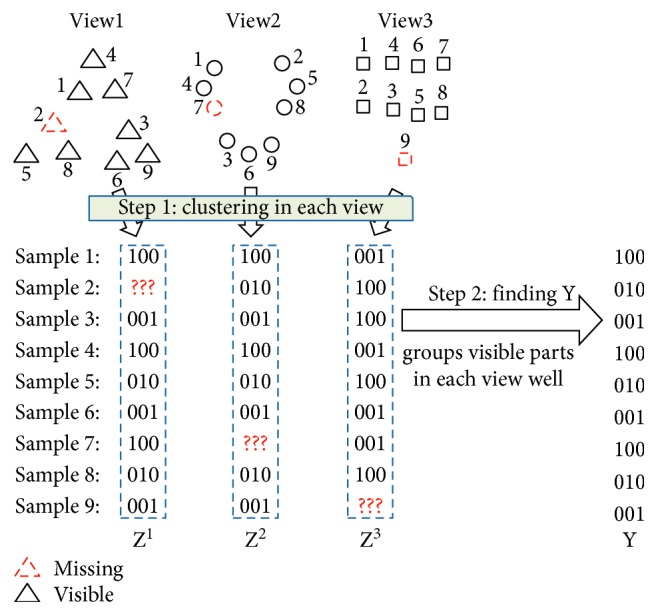
A brief example of incomplete multiview clustering via late fusion. **Z**^1^, **Z**^2^, and **Z**^3^ are clustering results from three views and **Y** is the final clustering.

Since clustering results from incomplete views could thus be made reliable, this enables us to propose a late fusion method for incomplete multiview clustering, while most of the previous studies focus on early fusion methods. Firstly, we perform kernel *k*-means clustering on the visible instances in each view. The clustering result of each view is encoded as a zero-one indicator matrix, each row of which contains the label information of the corresponding instance. Since some instances may be missing in some views, the corresponding rows of the matrices of some views may also be missing. These indicator matrices can also be considered as compressed representations of different views. Secondly, to create a fusion of the clustering results from different views, we develop an algorithm to find a clustering decision that can group each view's visible compressed representations well according to *k*-means objectives.  Figure 3 presents the process of the proposed method along with a brief example. Compared with several imputation-based methods and a representative early fusion method, the proposed method has superior clustering performance.

We conclude this section by highlighting the main contributions of this work, as follows: (1) We propose a late fusion method for incomplete multiview clustering, while most previous studies have concentrated on early fusion methods. Experimental results also validate the effectiveness of the proposed method. (2) In the second step of the proposed method, we design an alternate updating algorithm with proved convergence to learn the clustering decision that achieves the best *k*-means objective values with the visible instances in each view. (3) We provide some practical advice on initializing the clustering decision via analyzing the results of the comprehensive experiments.

## 2. Preliminary

In this section, we introduce some preliminary knowledge to facilitate better understanding of our proposed method. We first outline the notations used in this paper, after which *k*-means clustering and kernel *k*-means clustering are briefly reviewed, since these methods will be used in the proposed late fusion method.

### 2.1. Notation

Suppose the incomplete multiview data have *N* samples and *P* views. A sample should have at least one visible view. A sample's representation in a view, which is a row vector, is called an instance. Suppose the instances in view *j* are row vectors with length *d*_*j*_, which means the instances in view *j* have *d*_*j*_ features. Thus, the instances in view *j* form a *N* × *d*_*j*_ matrix, which is denoted as **X**^*j*^. Accordingly, we use **X**_*j*_^*i*^ to denote the instance for sample *i* in view *j*. An *N* × *P* zero-one matrix **S** stores the view missing information, where **S**_*ij*_=1 indicates that view *j* for sample *i* is available; otherwise, the view is missing. Assume that the actual number of clusters, denoted as *K*, is already known. We can thus perform clustering in each view *j*. An indicator matrix **Z**^*j*^ ∈ {0,1}^*N*×*K*^ is used to store the clustering result. If the instance of sample *i* is missing in view *j*, the *i*th row of **Z**^*j*^ is all zero; otherwise, if sample *i* belongs to cluster *c* in view *j*, we have **Z**_*ic*_^*j*^=1 and **Z**_*ik*_^*j*^=0, *k* ≠ *c*. The goal of incomplete multiview clustering is to find a clustering decision from all views. Similarly, we use a zero-one *N* × *K* matrix **Y** to store the clustering decision.

### 2.2. *k*-Means Clustering

The idea behind *k*-means clustering is to find a clustering assignment and a set of cluster centroids that bring the samples in each cluster closer to the corresponding centroid. Sum-of-squares loss is minimized to achieve this goal. Assume that {**x**_*i*_}_*i*=1_^*N*^ ∈ *𝒳* is the sample set and **Z** ∈ {0,1}^*N*×*K*^ is the unknown cluster indicator matrix, where **Z**_*ic*_=1 means that sample *i* belongs to cluster *c*. *μ*_*c*_ is the centroid of cluster *c*. The objective function of *k*-means is

(1) $$\begin{array}{c} \underset{Z,{\left\{\mu_{c}\right\}}_{i=1}^{K}}{\min}\;\overset{K}{\underset{c=1}{\sum }}\overset{N}{\underset{i=1}{\sum }}Z_{ic}{\left\|x_{i}-\mu_{c}\right\|}_{2}^{2},\\ \text{s}.\text{t}.\;\overset{K}{\underset{c=1}{\sum }}Z_{ic}=1,Z\in {\left\{0,1\right\}}^{N\times K}. \end{array}$$

An alternate updating algorithm is designed to solve this problem. Firstly, the centroids of the clusters are initialized. The cluster assignment is then updated by assigning the cluster label of each sample according to the closest centroid. Next, the centroids are updated by calculating the average of the samples in each cluster. The centroids and the cluster assignment are alternately updated until the cluster assignment no longer changes.

### 2.3. Kernel *k*-Means Clustering

Kernel *k*-means clustering is the kernel version of *k*-means clustering [15]. The objective is to find a cluster assignment that minimizes the sum-of-squares loss between the samples and the corresponding centroids in the kernel space. The kernel mapping from *𝒳* to a reproducing kernel Hilbert space *ℋ* is *ϕ*(·) : **x** ∈ *𝒳*⟶*ℋ*. The objective of kernel *k*-means clustering is as follows:

(2) $$\begin{array}{c} \underset{Z}{\min}\;\overset{K}{\underset{c=1}{\sum }}\overset{N}{\underset{i=1}{\sum }}Z_{ic}{\left\|\phi \left(x_{i}\right)-\mu_{c}\right\|}_{2}^{2},\\ \text{s}.\text{t}.\;\overset{K}{\underset{c=1}{\sum }}Z_{ic}=1,Z\in {\left\{0,1\right\}}^{N\times K}, \end{array}$$

where *μ*_*c*_=(1/*N*_*c*_)∑_*i*=1_^*N*^**Z**_*ic*_*ϕ*(**x**_*i*_) is the centroid of cluster *c* and *N*_*c*_=∑_*i*=1_^*N*^**Z**_*ic*_ is the number of samples in cluster *c*.

Define **K** satisfies *K*_*ij*_=*ϕ*(*x*_*i*_)^*T*^*ϕ*(*x*_*j*_) and **L**=diag([*N*_1_^−1^, *N*_2_^−1^,…, *N*_*k*_^−1^]). tr(·) is the trace operator and 1_*K*_ is an all-one column vector with length *K*. The equivalent matrix form of Equation (2) is

(3) $$\begin{array}{c} \underset{Z}{\min}\;\text{tr}\left(K\right)-\text{tr}\left(L^{1/2}Z^{T}KZL^{1/2}\right),\\ \text{s}.\text{t}.\;Z1_{K}=1_{N},Z\in {\left\{0,1\right\}}^{N\times K}. \end{array}$$

However, the problem in Equation (3) is difficult to solve due to the discrete constraint on variable **Z**. Accordingly, we may instead solve an approximated problem where **Z** is relaxed to real values. Letting **U**=**ZL**^1/2^ leaves us with the following problem:

(4) $$\begin{array}{c} \underset{U\in R^{N\times K}}{\max}\;\text{tr}\left(U^{T}KU\right),\\ \text{s}.\text{t}.\;U^{T}U=I_{K}, \end{array}$$

where the constant tr(**K**) is removed. The optimal **U** is found by calculating the *K* eigenvectors that correspond to the *K* largest eigenvalues of **K**. Since **U** can serve as a projection of the samples to space *ℝ*^*K*^, *k*-means clustering is performed on **U** to obtain the final cluster assignment.

## 3. The Proposed Method

In a departure from conventional subspace methods, we develop a late fusion method for incomplete multiview clustering. This method performs kernel *k*-means clustering in each incomplete view and then finds a consensus cluster according to each view's clustering result. The first step of the late fusion method, which is easy to understand, will be introduced only briefly. We will focus primarily on the second step to explain how a fusion of the incomplete clustering results from different views might be created. The overall algorithm is then presented and its complexity analyzed.

### 3.1. Clustering with Visible Instances in Each View

In line with most of the previous research into incomplete multiview clustering, we also assume that the instances in each view satisfy the random missing assumption. Although there are missing instances in an incomplete view, a common clustering method can be applied directly to the visible instances. As pointed out in the introduction, the clustering results in each view are reliable, which makes the late fusion of these results promising. In this paper, we perform kernel *k*-means on each incomplete view, since the multiview datasets are kernel data. Another clustering method could also be used in this step. It should be noted that while different clustering methods may have different robustness to random missing conditions, an investigation of this is beyond the scope of this paper. The clustering results are encoded as zero-one matrices: {**Z**^1^, **Z**^2^,…, **Z**^*P*^}, as described in the Notation section.

### 3.2. The Proposed Late Fusion Objective

To create a fusion of the clustering results {**Z**^1^, **Z**^2^,…, **Z**^*P*^}, we consider these clustering results as compressed representations in each view. Each row of the matrix can also serve as a compressed representation of the corresponding instance. The aim is to find a final clustering decision that can adequately group the compressed representations in each view. For the incomplete view, it is natural to expect that the remaining visible parts of the view can also be grouped well according to the final clustering decision.

For view *j*, we use **Z**_*i*_^*j*^ to denote the *i*th row of **Z**^*j*^, while **Z**_*i*_^*j*^ is the cluster label for the *i*th instance in view *j*. However, **Z**_*i*_^*j*^ can also serve as a compressed representation of the *i*th instance. When performing clustering on **Z**^*j*^, suppose the cluster indicator matrix is **Y** and the centroid of cluster *c* is **M**_*c*_^*j*^. The objective function for performing *k*-means clustering with the visible compressed representations in view *j* is thus

(5) $$\begin{array}{c} \underset{Y^{j},{\left\{M_{c}^{j}\right\}}_{c=1}^{K}}{\min}\;\overset{K}{\underset{c=1}{\sum }}\overset{N}{\underset{i=1}{\sum }}Y_{ic}^{j}S_{ij}{\left\|Z_{i}^{j}-M_{c}^{j}\right\|}_{2}^{2},\\ \text{s}.\text{t}.\;\overset{K}{\underset{c=1}{\sum }}Y_{ic}^{j}=1,\;Y^{j}\in {\left\{0,1\right\}}^{N\times K}, \end{array}$$

where **S**_*ij*_ is used to select the visible parts following the description in the Notation section.

For the multiview situation, we wish to find a consistent clustering decision **Y** that groups each view's visible compressed representations adequately. Thus, we propose to minimize the sum of the *k*-means objective values of all views with the visible compressed representations. The proposed objective function is as follows:

(6) $$\begin{array}{c} \underset{Y,{\left\{M_{c}^{j}\right\}}_{c=1j=1}^{K\;P}}{\min}\;\overset{P}{\underset{j=1}{\sum }}\overset{K}{\underset{c=1}{\sum }}\overset{N}{\underset{i=1}{\sum }}Y_{ic}S_{ij}{\left\|Z_{i}^{j}-M_{c}^{j}\right\|}_{2}^{2},\\ \text{s}.\text{t}.\;\overset{K}{\underset{c=1}{\sum }}Y_{ic}=1,\;Y\in {\left\{0,1\right\}}^{N\times K}. \end{array}$$

### 3.3. Optimization of the Late Fusion Objective

Similar to *k*-means clustering, we iteratively update **Y** and {**M**_*c*_^*j*^}_*c*=1_^*K*^ to solve the problem in Equation (6).

(1) *Updating ***Y**: when {**M**_*c*_}_*c*=1_^*K*^ are fixed, the optimization problem is
  (7) $$\begin{array}{c} \underset{Y}{\min}\;\overset{N}{\underset{i=1}{\sum }}\overset{K}{\underset{c=1}{\sum }}Y_{ic}\overset{P}{\underset{j=1}{\sum }}S_{ij}{\left\|Z_{i}^{j}-M_{c}^{j}\right\|}_{2}^{2},\\ \text{s}.\text{t}.\;\overset{K}{\underset{c=1}{\sum }}Y_{ic}=1,Y\in {\left\{0,1\right\}}^{N\times K}. \end{array}$$

The updating of **Y** is similar to that of the *k*-means clustering:

(8) $$\begin{array}{c} Y_{ic}=\left\{\begin{array}{cc} 1, & \text{if }c\text{ minimizes}\overset{P}{\underset{j=1}{\sum }}S_{ij}{\left\|Z_{i}^{j}-M_{c}^{j}\right\|}_{2}^{2},\\ 0, & \text{else}. \end{array}\right. \end{array}$$

**Lemma 1 .**

Equation. (8) is the optimal solution for the optimization problem in Equation (7).

*Proof*. Minimizing Equation (7) is equivalent to minimizing the following subproblem separately:

(9) $$\begin{array}{c} \underset{{\left\{Y_{ic}\right\}}_{c=1}^{K}}{\min}\;\overset{K}{\underset{c=1}{\sum }}Y_{ic}\overset{P}{\underset{j=1}{\sum }}S_{ij}{\left\|Z_{i}^{j}-M_{c}^{j}\right\|}_{2}^{2},\\ \text{s}.\text{t}.\;\overset{K}{\underset{c=1}{\sum }}Y_{ic}=1,Y_{ic}\in {\left\{0,1\right\}}^{N\times K}. \end{array}$$

Denoting *G*_*ic*_=∑_*j*=1_^*P*^**S**_*ij*_‖**Z**_*i*_^*j*^ − **M**_*c*_^*j*^‖_2_^2^, we then have

(10) $$\begin{array}{c} \overset{K}{\underset{c=1}{\sum }}Y_{ic}G_{ic}\geq \min{\left\{G_{ic}\right\}}_{c=1}^{P}\overset{K}{\underset{c=1}{\sum }}Y_{ic}=\min{\left\{G_{ic}\right\}}_{c=1}^{P}. \end{array}$$

When **Y**_*ic*_ follows Equation (8), according to Equation (10), ∑_*c*=1_^*K*^**Y**_*ic*_*G*_*ic*_ reaches its minimum.

(2) *Updating ***M**: when **Y** is fixed, the optimization problem is
  (11) $$\begin{array}{c} \underset{{\left\{M_{c}\right\}}_{c=1}^{K}}{\min}\overset{K}{\underset{c=1}{\sum }}\overset{N}{\underset{i=1}{\sum }}Y_{ic}\overset{P}{\underset{j=1}{\sum }}S_{ij}{\left\|Z_{i}^{j}-M_{c}^{j}\right\|}_{2}^{2}. \end{array}$$

By taking the derivative of Equation (11) with respect to **M**_*c*_^*j*^ to be 0, we can obtain the updated **M**_*c*_^*j*^ as

(12) $$\begin{array}{c} M_{c}^{j}=\frac{\sum_{i=1}^{N}Y_{ic}S_{ij}Z_{i}^{j}}{\sum_{i=1}^{N}Y_{ic}S_{ij}}. \end{array}$$

**Lemma 2 .**

Equation (12) is the optimal solution for the optimization in Equation (11).

*Proof*. Equation (11) is equivalent to

(13) $$\begin{array}{c} \underset{{\left\{M_{c}^{j}\right\}}_{c=1j=1}^{K\;P}}{\min}\overset{K}{\underset{c=1}{\sum }}\overset{P}{\underset{j=1}{\sum }}\overset{N}{\underset{i=1}{\sum }}Y_{ic}S_{ij}{\left\|Z_{i}^{j}-M_{c}^{j}\right\|}_{2}^{2}. \end{array}$$

Therefore, to minimize Equation (11) is equivalent to minimize **M**_*c*_^*j*^ separately. The subproblem of minimizing **M**_*c*_^*j*^ is as follows:

(14) $$\begin{array}{c} \underset{M_{c}^{j}}{\min}\overset{N}{\underset{i=1}{\sum }}Y_{ic}S_{ij}{\left\|Z_{i}^{j}-M_{c}^{j}\right\|}_{2}^{2}. \end{array}$$

The derivative of **M**_*c*_^*j*^ is as follows:

(15) $$\begin{array}{c} 2\overset{N}{\underset{i=1}{\sum }}Y_{ic}S_{ij}\left(Z_{i}^{j}-M_{c}^{j}\right), \end{array}$$

where **M**_*c*_^*j*^ is set as Equations (12) and (15) equals 0. Because Equation (14) is convex, Equation (14) reaches its minimum. Therefore, each subproblem reaches its minimum, meaning that Equation (11) also reaches its minimum.

### 3.4. Convergence of the Alternate Optimization

**Theorem 1 .**

The alternate updating of **Y** and {**M**_*c*_^*j*^}_*c*=1*j*=1_^*K* *P*^ converges.

*Proof*. According to Lemma 1 and Lemma 2, in the updating of both **Y** and {**M**_*c*_}_*c*=1_^*K*^, the objective value is not increasing. Moreover, because **Y** ∈ {0,1}^*N*×*K*^, **S** ∈ {0,1}^*N*×*P*^, and ‖**Z**_*i*_^*j*^ − **M**_*c*_^*j*^‖_2_^2^ ≥ 0, the objective value is lower bounded by 0. As a result, the alternate updating procedure converges.

### 3.5. Initialization for **Y**

For the alternate optimization, **Y** should be initialized in order to begin the optimizing process. The initialization of **Y** is an important factor in the performance of the final clustering decision. In order to obtain better performance, the initialization is not random. Instead, we use a basic method for incomplete multiview clustering to obtain an initial indicator matrix **Y**^0^. For example, we can first fill the incomplete data with a filling method such as zero-filling and then perform multiple kernel *k*-means clustering to obtain an initial indicator matrix **Y**^0^. Selecting a suitable method to obtain **Y**^0^ is crucial for the proposed method. We will explore this through a number of experiments in the Experiments section.

### 3.6. The Proposed Algorithm and Complexity Analysis

The overall algorithm is summarized in Algorithm 1. When learning the clustering results from each view, the initialization of {**M**_*c*_^*j*^}_*c*=1*j*=1_^*K* *P*^ is an important factor that affects the performance of the final clustering decision. In order to obtain a better performance, the initialization is not random. Instead, we calculate {**M**_*c*_^*j*^}_*c*=1*j*=1_^*K* *P*^ following Equation (12) with an initial indicator matrix **Y**^0^ from another basic solution of incomplete multiview clustering. Again, choosing a suitable **Y**^0^ is crucial for the proposed method, and we will therefore explore this with comprehensive experiments in the following Experiments section.

Eigenvector decomposition is applied to solve the kernel *k*-means problem. The time complexity for eigenvector decomposition using the most popular QR algorithm is **O**(*N*^3^) [16]. For all views, the complexity is **O**(*PN*^3^). Assume that the alternate updating procedure iterates *R* times. For each iteration, the complexity of updating **Y** is **O**(*PNK*^2^), while according to Equation (12), the complexity of updating {**M**_*c*_}_*c*=1_^*K*^ is **O**(*PNK*). Accordingly, the overall complexity of the proposed late fusion is **O**(*PN*^3^+*RPNK*^2^).

## 4. Experiments

### 4.1. Datasets

Experimental comparisons are conducted on six multiple kernel learning benchmark datasets. In these datasets, each kernel serves as a view.

#### 4.1.1. Caltech102

A precomputed kernel dataset from [17], which is generated from the object categorization dataset Caltech101. This dataset can be downloaded from http://files.is.tue.mpg.de/pgehler/projects/iccv09/#download.

#### 4.1.2. CCV

Consumer video analysis benchmark dataset proposed in [18]. The original dataset can be downloaded form http://www.ee.columbia.edu/ln/dvmm/CCV/. We compute three linear kernels on its MFCC, SIFT, and STIP features and then compute three Gaussian kernels on these features, where widths are set as the mean of sample pair distances.

#### 4.1.3. Digital

Handwritten numerals (0–9) dataset from UCI Machine Learning Repository. The original dataset consists of 6 feature sets and can be downloaded from http://archive.ics.uci.edu/ml/datasets/Multiple+Features. Following the settings in [6], we select 3 of 6 feature sets (Fourier feature set, pixel averages feature set, and morphological feature set) to generate 3 kernels.

#### 4.1.4. Flower17

17 category flower dataset from Visual Geometry Group. The original dataset can be downloaded from http://www.robots.ox.ac.uk/~vgg/data/flowers/17/index.html.

#### 4.1.5. Flower102

102 category flower dataset from Visual Geometry Group. The original dataset can be downloaded from http://www.robots.ox.ac.uk/~vgg/data/flowers/102/index.html.

#### 4.1.6. ProteinFold

Fold recognition dataset which consists of 694 proteins with 27 SCOP fold [19]. Following the settings in [19], we generate 10 second order polynomial kernels and two inner product kernels. The matlab file of the kernel data can be downloaded from https://github.com/HoiYe/MKL_datasets/blob/master/proteinFold_Kmatrix.mat.

The basic information of these datasets is summarized in Table 1.

### 4.2. Compared Methods

The proposed method is compared with several imputation methods and a representative early fusion method. Moreover, the best result of a single view is also provided as a baseline.

#### 4.2.1. Best Result of a Single View (BS)

The best clustering result from a view. We select the view that has the highest clustering performance with the visible instances. If this view is incomplete, we assign the missing instances with random labels and then report the performance.

#### 4.2.2. Zero Filling Plus Multiple Kernel *k*-Means (ZF)

The missing kernel entries are filled by zero, after which multiple kernel *k*-means clustering is applied.

#### 4.2.3. Mean Filling Plus Multiple Kernel *k*-Means (MF)

The missing kernel entries are filled by the average value of the corresponding visible entries in other views. Multiple kernel *k*-means clustering is then applied.

#### 4.2.4. *k*-Nearest Neighbor Filling Plus Multiple Kernel *k*-Means (KNN)

The incomplete kernels are filled using the *k*-nearest neighbor imputation algorithm, after which multiple kernel *k*-means is applied.

#### 4.2.5. Alignment-Maximization Filling Plus Multiple Kernel *k*-Means (AF)

The alignment-maximization filling proposed in [11] is a simple yet efficient kernel imputation method. A complete kernel is generated by averaging the zero-filled kernels of each view, after which each incomplete kernel is filled with this complete kernel according to the algorithm in [11]. Multiple kernel *k*-means clustering is applied after filling the incomplete kernels.

#### 4.2.6. Partial View Clustering (PVC)

This subspace method, proposed in [20], tries to learn a subspace where two views' instances of the same sample are similar. It is a representative early fusion method for incomplete multiview clustering.

### 4.3. Experimental Setting

In our experiments, the number of clusters is considered as prior knowledge. Base kernels are centralized and scaled during the preprocessing procedure following the suggestion in [21].

Since the base kernels are complete in the original datasets, the incomplete kernels need to be generated manually. We assume that the ratio of samples with missing views (incomplete sample ratio) is *ϵ*. To generate the missing view information matrix **S**, we randomly select *ϵ* × *N* samples. The missing probability of a view is *q*_0_. Next, for each sample that has incomplete views, a random vector **g**=(**g**_1_,…, **g**_*P*_) ∈ [0,1]^*P*^ is generated. The *p*th view will be missing for this sample if **g**_*p*_ < *q*_0_. Since at least one view should exist for a sample, we will generate a new random vector until at least one view for the sample is present. In our experiments, ϵ varies from 0.1 to 0.9 to demonstrate how the performance of different methods varies with respect to *ϵ*, while *q*_0_ is fixed as 0.5. Normalized mutual information (NMI) is applied to evaluate the clustering performance.

### 4.4. Experimental Results

#### 4.4.1. Late Fusion Performance with Different Initializations

The proposed method requires an initial clustering decision **Y**^0^ for the late fusion process. In this paper, the clustering decision of other commonly used imputation methods is employed for initialization. We expect performance improvement after late fusion compared with the initial clustering decision. In Table 2, we compare the performance of the initial method and the corresponding late fusion result on six benchmark datasets with different incomplete sample ratios. The better performance value is shown in bold. It can be observed that improvements are evident in most situations under late fusion conditions. On ProteinFold, Flower17, Caltech102, and Digital, a consistent boost with late fusion can be achieved; for example, late fusion performance is about 27% higher compared with the BS initial result when 80% of samples are incomplete on Digital. The reason for this performance boost is that the late fusion step considers the consistency between views and leverages the information from both views to revise the initial clustering. However, there are some exceptions for performance improvement. On CCV, the late fusion result is worse than AF when 20% of samples are incomplete. We suggest that these results emerge for the following reasons: first, AF can achieve a fairly good imputation on CCV when the incomplete sample ratio is 20%; second, the views of CCV may not be highly consistent, which could hurt the efficiency of the late fusion step. When the incomplete sample ratio is 80%, use of late fusion fails to improve the performance for three of five methods on Flower102. This indicates that late fusion is also hurt when percentage of incomplete samples is high. Because the late fusion method is based on the consistency assumption, we can assume that the inclusion of some noisy views, due to the high incomplete sample ratio in Flower102, has attenuated the performance of the late fusion method. However, in most cases, the late fusion procedure's performance is improved relative to the initial method. Exceptions occur when the consistency between the views of the dataset is not strong or the initial method is highly effective. It is noteworthy that the late fusion procedure can be viewed as a refined progress for the initial method's clustering decision.

#### 4.4.2. Choosing Initialization Method

Although the experimental results in the previous section show that improvement can be obtained using the late fusion method, the question of how to choose a suitable initialization to ensure the best final performance remains unsolved. In this section, we conduct some empirical studies to determine the relationship between the initialization method and the final late fusion performance.

For each dataset, we calculate the mean NMI of different incomplete sample ratios for the late fusion of different initializations. Once this is complete, we rank the performance on each dataset to see which initialization achieve the best final performance, as shown in Table 3. Late fusion based on KNN ranks first on ProteinFold and Digital, while on Flower17 and Caltech102, late fusion based on AF achieves the best performance. On the two relatively large datasets, that is, CCV and Flower102, late fusion based on BS is most suitable. In the last two columns of Table 3, “rankScore” denotes the average rank of six datasets, while “overall” denotes the rank of “rankScore.” The “overall” column indicates that AF may be a good choice for the best final fusion performance over the six datasets.

However, as shown in Table 2, it is possible that late fusion performance could be worse than the initial result. Therefore, we also investigate the relative late fusion performance changes of different initializations to see which initial methods may be boosted less via late fusion. Similarly, for each dataset, we calculate the mean NMI changes of different incomplete ratios after late fusion for different initialization.  Table 4 shows that BS, ZF, and MF benefit substantially from late fusion; for example, when using BS as initialization, there is an 18.09% boost on Digital. However, the late fusion method cannot make a significant boost against AF, as the boost on Digital is only small at 6.47%.

In short, it may be impossible to find a universal best initialization technique for the proposed late fusion method. However, the empirical results allow us to draw some conclusions regarding the choice of initialization. (1) If we have a strong prior knowledge to decide which view is most important, BS may be a suitable initialization, since BS can be substantially boosted by late fusion (overall rank 1 as shown in Table 4) and achieves relatively good final performance (rank 1 on Digital and CCV, final rank 4 as shown in Table 3). (2) Although AF is a very good initialization that leads to the best late fusion performance (overall rank 1 as showed in Table 3), there is a risk that the late fusion process may not give better results than the initial one. (3) ZF and MF are not recommended to be used as initializations, due to their poor final late fusion performance.

#### 4.4.3. Comparisons between the Best Late Fusion and the Basic Methods

**Figure 4:**
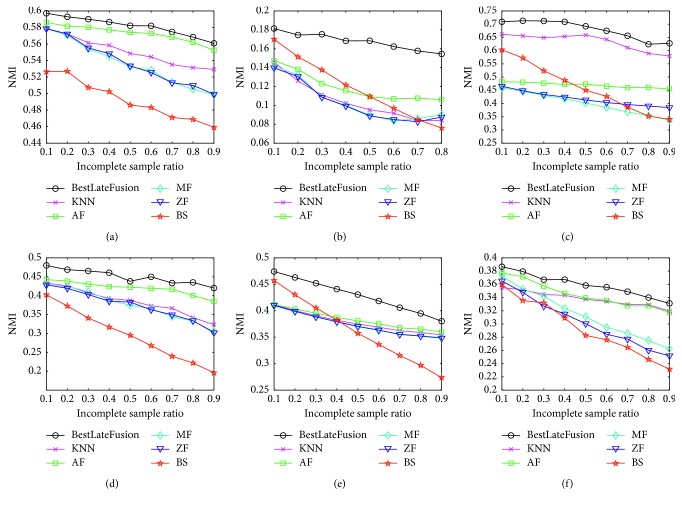
Comparison between the best late fusion and the commonly used imputation methods. (a) Performance on Caltech102. (b) Performance on CCV. (c) Performance on Digital. (d) Performance on Flower17. (e) Performance on Flower102. (f) Performance on ProteinFold.

Figure 4 shows that when the best initialization is used, the performance of the proposed late fusion method can always achieve the best NMI on the six benchmark datasets compared with basic methods. For example, on the challenging dataset CCV, the performance of late fusion with the best initialization outperforms other methods in different incomplete sample ratios. More specifically, when incomplete ratio is 0.9, the late fusion method significantly outperforms the second best method by around 5%. The results in Figure 4 indicate that the proposed late fusion method can benefit from a suitable initialization to achieve better performance than the commonly used imputation methods.

#### 4.4.4. Comparisons with Early Fusion Method for Two Views

**Figure 5:**
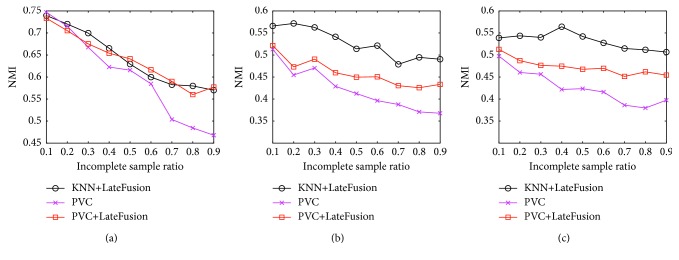
Comparison with early fusion method. (a) Performance on Digital view 1 and view 2. (b) Performance on Digital view 1 and view 3. (c) Performance on Digital view 2 and view 3.

In this section, we compare the proposed method with partial view clustering (PVC), which is a representative early fusion method proposed in [20]. PVC is a method originally designed for two views, such that it is difficult to adapt it to more than two views. Therefore, we compare the performance on two views selected from Digital. According to the experimental results presented in Table 3, KNN is the best initialization on Digital; we thus compare the performance of PVC with late fusion using KNN as the initial method. Moreover, we compare the result of late fusion with the PVC initialization to determine whether the late fusion method can boost the performance of the initial PVC clustering decision. From Figure 5, we can observe that the late fusion step can result in an improvement over using PVC as the initial method, since PVC + ate fusion always has better performance than PVC. On view 1 and view 2, the performance of PVC + late fusion is comparable with KNN + late fusion.The result of view 1 and view 3 and the result of view 2 and view 3 show that KNN + late fusion has the best performance, and significantly outperforms PVC. Overall, the results on Digital indicate that the proposed late fusion method can improve the PVC clustering decision and can also outperform PVC significantly with suitable initialization. On a note of particular interest, the results indicate that the proposed late fusion process can refine the results of the early fusion method.

## 5. Conclusion

In this paper, we propose a novel late fusion method to learn a consensus clustering decision from the clustering results of incomplete views without imputation. To learn the consensus clustering decision, we design an alternate updating algorithm and prove its convergence theoretically. Moreover, we perform comprehensive experiments to study carefully how the initialization affects the final performance of the proposed method. Although we cannot find a best initialization for all situations, we suggest that the clustering result of the best single view is an effective initialization. With suitable initialization, the proposed method outperforms the commonly used imputation methods and a representative early fusion method.

Although the proposed method demonstrates the effectiveness of late fusion strategy in the field of incomplete multiview clustering, there are several promising directions for further research. First direction is to automatically generate the clusters without fixing the number of clusters. In many real-world applications of clustering, the number of clusters is unknown, where the proposed method cannot be applied. Instead of using kernel *k*-means clustering, we can perform other density-based clustering methods to get the clustering result in single view [22] and then design a new method to integrate the information between views. To integrate the density-based clustering results is a challenging problem. Second direction is to apply deep learning techniques for better late fusion results. Since 3DConvNets has achieved great success in feature learning [23], performing late fusion after feature learning with 3DConvNets may improve the final clustering performance. Third direction is to investigate how the clustering method in single view can affect the late fusion performance. In this paper, we perform kernel *k*-means clustering in each incomplete view. However, there are also other optional advancing clustering methods [24–27]. What kind of methods is suitable for late fusion for incomplete multiview clustering remains unrevealed.

## Figures and Tables

**Algorithm 1:**
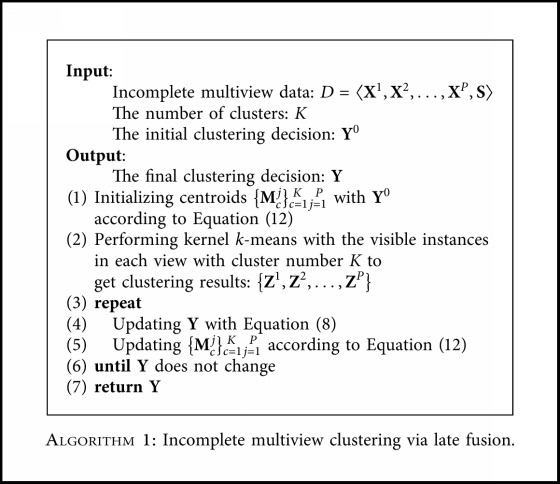
Incomplete multiview clustering via late fusion.

**Table 1:** Information of datasets.

Dataset	Sample number	Kernel number	Cluster number
Caltech102	1530	25	102
CCV	6773	6	20
Digital	2000	3	10
Flower17	1360	7	17
Flower102	8189	4	102
ProteinFold	694	12	27

**Table 2:** Performance comparisons between the initialization and the corresponding late fusion in terms of NMI (%).

	ProteinFold		Flower17		Caltech102		Digital		CCV		Flower102	
	Initial	Late fusion	Initial	Late fusion	Initial	Late fusion	Initial	Late fusion	Initial	Late fusion	Initial	Late fusion
*Incomplete sample ratio 20%*												
BS	33.53	**36.93**	37.27	**44.26**	52.70	**55.11**	57.24	**66.08**	15.14	**17.45**	43.05	**46.30**
ZF	34.79	**37.96**	41.95	**46.39**	57.21	**58.86**	44.85	**54.98**	13.06	**13.09**	39.90	**40.50**
MF	35.27	**37.87**	42.45	**47.23**	57.07	**58.84**	44.56	**51.60**	13.23	**13.36**	39.83	**40.40**
KNN	35.19	**37.94**	42.60	**46.42**	57.23	**58.92**	65.59	**71.30**	12.59	**13.34**	40.04	**40.44**
AF	37.14	**38.89**	43.87	**46.88**	58.16	**59.30**	48.08	**54.40**	**13.82**	13.20	40.35	**40.51**

*Incomplete sample ratio 50%*												
BS	28.27	**34.20**	39.52	**43.64**	48.62	**52.96**	45.03	**63.84**	10.94	**16.84**	35.74	**43.06**
ZF	30.05	**34.14**	38.28	**44.54**	53.35	**56.17**	41.34	**51.87**	8.88	**12.82**	37.04	**38.02**
MF	31.10	**34.15**	37.45	**44.02**	53.26	**56.33**	40.17	**49.91**	8.97	**12.76**	36.98	**38.00**
KNN	33.73	**35.83**	38.74	**44.42**	54.86	**56.80**	65.90	**69.19**	9.53	**12.73**	37.52	**38.09**
AF	33.94	**35.68**	42.24	**43.80**	57.41	**58.22**	47.35	**53.98**	10.92	**12.64**	38.08	**38.18**

*Incomplete sample ratio 80%*												
BS	24.63	**32.58**	22.18	**42.51**	46.87	**51.36**	35.28	**62.27**	7.61	**15.45**	29.66	**39.49**
ZF	26.03	**30.29**	33.38	**42.79**	50.99	**54.04**	39.06	**51.51**	8.76	**12.62**	35.21	**35.23**
MF	27.50	**30.92**	33.28	**42.63**	50.53	**53.75**	35.38	**48.74**	9.01	**12.98**	**35.27**	35.17
KNN	32.92	**33.99**	34.18	**41.64**	53.14	**54.95**	58.95	**62.43**	8.40	**12.59**	**35.90**	35.34
AF	32.73	**33.73**	40.02	**43.51**	56.21	**56.84**	46.07	**52.58**	10.61	**12.93**	**36.53**	35.66

**Table 3:** Rank of the late fusion performance with different initializations in terms of NMI (%).

	ProteinFold		Flower17		Caltech102		Digital		CCV		Flower102		Rank score	Overall
	Mean	Rank	Mean	Rank	Mean	Rank	Mean	Rank	Mean	Rank	Mean	Rank		
BS	34.62	3	43.67	5	53.02	5	64.28	2	16.78	1	42.89	1	3.33	3
ZF	34.18	5	44.70	2	56.22	3	52.82	4	12.80	5	37.95	4	4.83	4
MF	34.50	4	44.65	3	56.19	4	50.26	5	12.91	3	37.91	5	5.00	5
KNN	35.93	1	44.28	4	56.87	2	68.63	1	12.80	4	37.97	3	3.17	2
AF	35.90	2	45.02	1	58.17	1	53.77	3	13.11	2	38.14	2	2.50	1

**Table 4:** Rank of the performance change with different initializations on different datasets in terms of NMI (%).

	ProteinFold		Flower17		Caltech102		Digital		CCV		Flower102		Rank score	Overall
	Change	Rank	Change	Rank	Change	Rank	Change	Rank	Change	Rank	Change	Rank		
BS	5.31	1	14.20	1	3.78	1	18.09	1	4.93	1	6.73	1	1.00	1
ZF	3.86	2	7.29	2	2.48	3	10.87	2	2.52	2	0.55	2	2.17	2
MF	3.16	3	7.17	3	2.56	2	10.14	3	2.51	3	0.51	3	2.83	3
KNN	2.11	4	5.93	4	1.76	4	4.62	5	2.30	4	0.14	4	4.50	4
AF	1.47	5	3.02	5	0.89	5	6.47	4	1.18	5	−0.17	5	5.00	5

## Data Availability

The data used to support the findings of this study are available from the corresponding author upon request.
